# Breast Cancer Screening in Low-Income Countries: A New Program for Downstaging Breast Cancer in Tanzania

**DOI:** 10.1155/2022/9795534

**Published:** 2022-04-05

**Authors:** Darcy S. Cherlin, Julius Mwaiselage, Khadija Msami, Zoe Heisler, Heather Young, Xingwei Cui, Amr S. Soliman

**Affiliations:** ^1^Ocean Road Cancer Institute, Dar es Salaam, Tanzania; ^2^CUNY Graduate School of Public Health and Health Policy, The City College of New York, New York City, New York, USA; ^3^Department of Epidemiology and Biostatistics, Milken Institute School of Public Health, George Washington University, Washington, D.C., USA; ^4^Community Health and Social Medicine Department, CUNY School of Medicine, The City College of New York, New York City, New York, USA; ^5^Department of Global Health Epidemiology and Disease Control, Milken Institute School of Public Health, George Washington University, Washington, D.C., USA

## Abstract

**Background:**

Most breast cancer diagnoses in Tanzania are in advanced stages. The Ocean Road Cancer Institute (ORCI) established a new breast cancer screening program in 2014 to reduce advanced-stage diagnoses. This study is aimed at describing the screening program's referral process and at identifying patient and health system factors that contribute to patients completing diagnostic testing referrals.

**Methods:**

Six-hundred and forty patients were included in the study. Testing types, outcomes, and date of diagnostic results were abstracted from records at ORCI and Muhimbili National Hospital (MNH) to determine the proportion of testing completed and the duration between initial referrals and diagnostic tests. Prediction of completion of diagnostic testing was investigated in logistic regression.

**Results:**

Of the patients who received referrals for further testing, fifty-two percent completed the recommended ultrasound (USS), mammography (MMG), and fine-needle aspiration cytology (FNAC). Only 33.0% of patients completed the recommended MMG referrals compared to 55.0% for ultrasound and 68.7% for FNAC. The average number of days between initial screening and results was 42 days for MMG, 20 days for USS, and 18 days for FNAC. Significant predictors for completing referrals for USS, FNAC, and MMG included age < 44 and >55 years, presenting with symptoms at the initial appointment, and education. The odds of completing an USS was 3.03 (95% CI, 1.65-5.64) for patients 25–34, 2.27 (95% CI, 1.17-4.48) for patients 35–44, and 4.41 (95% CI, 1.66-10.11) for patients older than 55 years compared to the reference group (age 19–24). The presence of symptoms at the initial appointment was a significant predictor of FNAC. The odds of completing an FNAC was 1.55 (95% CI, 1.02-3.72) for symptomatic compared to nonsymptomatic patients. Education was a significant predictor of MMG. The odds of receiving MMG was 4.29 (95% CI, 1.05-21.00) for patients with tertiary education or higher compared to primary education or lower. Possession of health insurance for treatment and living in Dar es Salaam were not significant predictors. *Discussion.* Future research should focus on patients' understanding of recommended referrals and factors that influence decision-making. Investigating the cost effectiveness of scaling up screening programs and setting up a patient navigation program that follow patients as they complete the recommended treatment plan will be crucial for Tanzania and other developing countries as they seek to launch and strengthen screening programs.

## 1. Introduction

Breast cancer is the most common cancer among women worldwide with 1.7 million new cases diagnosed each year. Although developing countries experience lower incidence rates compared to developed countries, 62% of global deaths due to breast cancer occur in developing countries [1].

In Tanzania, a developing country, breast cancer is the second most common cancer and the second leading cause of cancer mortality (after cervical cancer). Previous research from Tanzania showed that more than 90% of breast cancers are diagnosed at advanced disease stages (stages III and IV) [2]. It was projected in the 2017 Health Assessment that Tanzania will see an increase in new breast cancer cases by 82% by 2030 [1]. This assessment concluded that inefficient clinical pathways for women with breast cancer cause significant delays in the detection, diagnosis, and treatment processes. Recommendations to ensure the effective continuum of care for patients included implementing referral protocols and patient tracking [1].

Currently, there is no national screening program in Tanzania. It has been recommended, in countries with low resources, that a streamlined cancer diagnostic and treatment infrastructure take place first before implementing widespread population-based screening [3].

ORCI, the largest and only cancer center with chemotherapy and radiotherapy treatment facilities, established a new breast cancer screening program in 2014 to reduce advanced-staged diagnoses. This study is aimed at describing the screening program's referral process and at identifying patient and health system factors that contribute to patients completing diagnostic testing referrals.

## 2. Material and Methods

### 2.1. Breast Cancer Screening Program

The Ocean Road Cancer Institute is the national cancer center of Tanzania, located in Dar es Salaam, the largest city of Tanzania. The population of Tanzania is approximately 62 million, and the population of Dar es Salaam is approximately 6 million [4, 5]. Women from all over Tanzania come to ORCI for cancer care [6–8].

The newly developed screening program includes a clinical breast exam (CBE) at the breast cancer screening clinic at ORCI and further diagnostic testing and treatment that can be completed at ORCI and Muhimbili National Hospital (MNH). The two institutes are located three miles (4.4 km) apart. The target population for the screening program is all women living in Dar es Salaam who are 40 years or older, which we would approximate to be about 270,000 women based on the Tanzania's population age structure [9].

This study took place at the newly developed breast cancer screening clinic at ORCI. The patient population of the clinic includes both self-referred patients and referrals from the well-established cervical screening clinic of ORCI that has been in operation since 2001 [7]. The referral process at the cervical screening clinic is spontaneous. Patients are referred either by word of mouth or through the healthcare system. Patients with suspected lesions are referred to ORCI from other clinics as shown in previous studies [6–8]. The breast cancer screening clinic can currently accommodate up to thirty-five women per day but the number is expected to increase to up to 100 women per day with the plans for increasing the number of physicians and nurses of the clinic.

**Figure 1:**
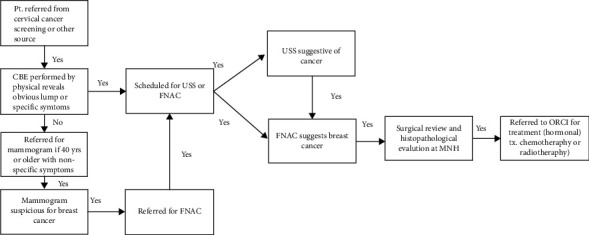
Pathway for patients seen at the ORCI breast cancer screening clinic who receive diagnostic testing referral. ^∗^Surgical review could involve an excisional or incisional biopsy, lumpectomy, partial mastectomy, or modified radical mastectomy.

At the breast cancer screening clinic, patients are given a clinical breast exam by a nurse and referred to a physician, if the exam is abnormal. The physician refers patients with abnormal findings for further diagnostic testing, either an ultrasound (USS) or fine needle aspiration and cytology with USS guide (FNAC), which are both performed at ORCI. There is currently no mammogram (MMG) at ORCI. Therefore, if the patients have nonspecific symptoms and are forty years or older, they are referred for an MMG at MNH or a different off-site facility. If patients have a positive MMG, they are referred back to ORCI for an FNAC. Patients with a FNAC suggestive of breast cancer are referred for histopathological examination (HPE) and surgical review at MNH, which could involve an excisional or incisional biopsy, lumpectomy, partial mastectomy, or modified radical mastectomy. If the test confirms cancer, these patients are referred to ORCI for treatment which could involve chemotherapy, hormonal treatment, or radiotherapy. Patients who have a positive FNAC that is not defined as suspicious for breast cancer (i.e., cyst or abscess) are either referred for further diagnostic review at MNH or a different diagnostic facility in Tanzania. The referral pathway described above is displayed in Figure 1.

### 2.2. Study Population and Data Collection

The study population included patients aged 19 and older, who attended the screening program and were referred for further diagnostic testing after a clinical breast exam from January 1st, 2018–December 31st, 2018. The data collection for this study was conducted during the period of May–August 2019. A search was run in the ORCI electronic medical record (EMR) system that identified 3,993 women who had a clinical breast exam screening at ORCI in 2018. We reviewed 3,993 records to identify patients referred for further diagnostic testing.

The referral for further diagnostic testing could be identified in medical records in the following ways: (1) referral notated in the management plan of the EMR, (2) a referral entered in the further testing portion of the EMR, (3) a diagnostic test ordered in the medical records, (4) recorded results from the diagnostic test, or (5) information available in the paper medical records housed on the treatment side of ORCI.

**Figure 2:**
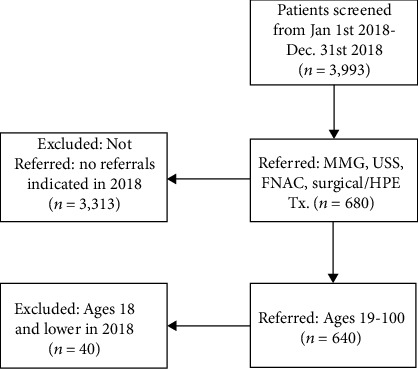
Inclusion and exclusion criteria of the study population.

Of the 3,993 women seen in the clinic for clinical breast exams in 2018, 640 women met the age and referral criteria (19 and older and referred for diagnostic testing after a CBE).  Figure 2 shows a flow diagram of the inclusion and exclusion process. Demographic information for the study participants including age, education status, insurance status, region, and districts of residence was collected from the profile section of each patient's EMR. The region and district of residence were used to construct a patient residence variable for living in Dar es Salaam and living outside of Dar es Salaam. Whether or not a patient reported symptoms at the initial breast clinical exam screening was also abstracted from the documentation of the initial visit in the EMR. Examples of symptoms at initial screening included lump, mass breast pain, breast tenderness, or nipple discharge. A patient was considered to have reported no symptoms if no symptoms were written or indicated in the medical record for that visit.

Further information abstracted from the medical records included the date of initial CBE, symptoms at presentation, and type of testing referral (USS, MMG, or FNAC) at the initial visit. The results and date of the results of the MMG and USS were collected from the EMRs. If a patient was referred for an MMG or USS and no results appeared in the medical record, this was categorized as “no test completed.” If results were reflected in the medical record, this was categorized as “completed test.” In the case that there was no date, the date was recorded as missing.

If a participant was referred for an FNAC at ORCI, the results and date of the reported results were collected from the electronic files created by the pathologists who completed the test. The date and results of each test were housed in individual electronic files titled by the first and last names of each patient. We searched for the FNAC results using first and last names. If there was no electronic file for the patient, this was categorized as “no test completed.” If there was an electronic file with the results, this was categorized as “completed test.”

### 2.3. Data Management and Statistical Analysis

Six hundred and forty participants were separated into two groups to assess the completion of diagnostic testing. The two groups included patients who completed and did not complete the test for USS, MMG, and FNAC. Patients who had only primary and no education were combined as the lowest level of education to be the reference group for education status for the regression model. Having used no insurance (cash private) was the reference group for insurance status, and the age range 19–24 served as the reference group for age. The odds of completing recommended follow-up testing was analyzed by age, education status, location of residence, symptoms at screening, and insurance status for those referred for a USS, MMG, or FNAC. A logistic regression model was constructed to model the odds of completion of testing as the dependent variable with age, education status, residence, clinical symptoms, and insurance status as independent factor predictors. Separate models were constructed for USS, MMG, and FNAC. Odds ratios and 95% confidence intervals were constructed. Chi-square and Fisher's exact tests were used as appropriate to determine *p* values. ArcGIS, a mapping analytics software, was used to further assess the impact of the geographical residence on the completion of diagnostic testing by displaying the two groups, those who did and did not have testing completed, by region of residence. All statistical analyses used RStudio and all spatial analyses used ArcGIS.

The study was approved by the Institutional Review Board of George Washington University and the Ethics Committee of Ocean Road Cancer Institute in Tanzania.

## 3. Results

Of the 3,993 patients screened for breast cancer in 2018, 640 patients between the ages of 19 and 100 were referred for further diagnostic testing. Within this group, there were 409 USS referrals, 118 MMG referrals, and 313 FNAC referrals. Of the patients referred, 55.0% completed USS testing, 33.9% completed mammogram testing, and 68.7% completed testing for an FNAC.

The average number of days from initial screening to when the results were recorded was 20 days for an USS, 42 days for an MMG, and 18 days for an FNAC. The minim number of days from the date of initial screening to the date that the results were recorded was 0 days for an USS, 1 day for an MMG, and 0 days for an FNAC. The maximums days were 268 days for an USS, 268 days for a MMG, and 201 days for an FNAC. There was a large range between the initial screening and date of reported results for each test (268 for USS, 267 for MMG, and 201 for FNAC).

The proportion of patients who did and did not complete the tests that they were referred for was stratified by age, education, symptoms at initial screening, residence, and insurance status (Tables 1–3).  Table 1 shows a fairly similar distribution between “completed test” and “no test completed” for an USS, for education (none or primary 41.8% vs. 50.3%, secondary 33.5% vs. 27.3%, and tertiary 24.7% vs. 22.4%), residence (does not live in Dar es Salaam 19.6% vs. 23.45, lives in Dar es Salaam 80.4% vs. 76.6%), insurance status (cash private 70% vs. 78.2%, NHIF 27% vs. 20.1%, and exempted 2.7% vs. 1.6%), and symptoms at initial referral (no symptoms 21.8% vs. 20.1%, symptoms 78.2% vs. 79.9%). A larger proportion of patients in the completed test group was between the ages 19–24 than that of the no completed test group, and a smaller proportion of patients in the “completed test” group was between ages 55+ compared to that of the “no test completed” group Table 2 shows little difference between the two groups for age, symptoms at initial referral, insurance status, and education level for the MMG test. A larger proportion of patients who did not live in Dar es Salaam was in the “no test completed” group compared to the “completed test” group. Table 3 shows more variation between the two groups for symptoms at initial referral, residence, and insurance status for the FNAC test. The age was similar across both groups.

Tables 4–6 show the crude and adjusted odds ratios stratified by age, education, symptoms at initial screening, residence, and insurance status.

Place of residence and insurance status were not statistically different between patients who did complete an USS, MMG, or FNAC and those who did not.

Age was found to be a significant predictor for completing the USS test in this regression model. The odds of completing an USS was 3.03 (95% CI, 1.65-5.64) times higher if a patient was between the ages of 25 and 34, 2.27 (95% CI, 1.17-4.48) times higher if a patient was between the ages of 35 and 44, and 4.41 (95% CI, 1.66-10.11) times higher if a patient was between the ages 55+ compared to the reference group (age 19–24). Age was a nonsignificant predictor for FNAC or MMG.

The presence of symptoms at initial referral was found to be a statistically significant predictor of the FNAC test. The odds of completing an FNAC was 1.55 (CI, 1.02-3.72) times higher if a patient had symptoms at screening compared with no symptoms at the screening. Symptoms at initial referral were a nonsignificant predictor for MMG or USS.

The education level was found to be a significant predictor for the MMG test. The odds of completing an MMG was 4.29 (CI, 1.05-21.00) times higher if a patient received tertiary education compared to none or primary education. Education was a nonsignificant predictor for USS and FNAC.

ArcGIS, a mapping and analytics software, was used to further evaluate the impact of the region of residence on receiving testing. The two groups, those who did and did not have completed, were displayed by the region of residence for each test. No significant pattern was found between the groups for USS, MMG, and FNAC.

Of the 604 patients in the study cohort, 46 patients were confirmed to have been diagnosed with cancer. This confirmation was based on the results of histopathology, available data from MNH, and/or returning to ORCI for treatment. The status of the remaining 558 women could not be determined becasue the women did not complete the diagnostic tests, their data were not availabel or missing, or women did not return back for treatment after the initial diagnosis. TNM data was used to estimate the AJCC stage. Of the 46 patients, 15.22% had stage 2a–2b, 63.04% had stage 3a–3c, 6.52% had stage 4, and 15.22% had no staging information available.

## 4. Discussion

Our study revealed the following interesting observations. First, patients completed about half the test referrals for further diagnostic investigations. Second, aging, education, and presenting symptoms of breast disease were significant predictors of receiving the diagnostic procedures. Third, insurance status and place of residence were not predictors of completing the recommended diagnostic investigations.

Regarding the first observation, our findings show that about 52% of referred study participants completed diagnostic tests. When comparing the degree of completion of all three diagnostic tests, a higher percentage of USS and FNAC test results suggests that more of these tests were completed by the patients referred to them compared to patients referred for MMG tests. Additionally, more time elapsed between the date of the initial screenings and the date that MMG results were recorded compared to USS or FNAC testing.

The program at ORCI requires patients to travel to MNH for MMG and HPE while USS and FNAC are provided at ORCI. The time needed for commuting to and from MNH and the time required for wait listing at MNH may have led to the lower rates of returning to ORCI with MMG results. A study that evaluated ORCI as a cancer institution found that having diagnosis and surgery completed at a different location led to “fragmented care,” which was a barrier to detection and management of breast cancer [1]. The evaluation study recommended that ORCI provides a full range of diagnostic, surgical, systemic, and radiation therapies and supportive care services to reduce these barriers. Another study from Mexico City found that the main barrier to health service utilization was access to care, especially referrals. Women described “exhaustion” from dealing with the process [10]. These findings suggest that having multiple sites for one screening program may be a barrier to completing diagnostic testing and treatment.

Regarding the second observation about the predictors of completing the diagnostic results, age was a significant predictor of completing an USS. Also, having symptoms at initial examination at ORCI and subsequent referral was substantial of completing an FNAC. Furthermore, the educational status was a significant predictor of completing an MMG.

The odds of completing an USS was found to be significantly higher in women 25–44 and 55+, compared to that of the reference group, ages 19–24. Age was a nonsignificant predictor of FNAC or MMG. We would like to emphasize that Tanzania, like most developing countries, has a significantly younger median age of the general population than the median age in developed countries. The median age in Tanzania is 18.2 compared to the US which has a median age of 38.5 [4]. Several studies found the mean age of breast cancer diagnosis in Tanzania to be 49–53 years [11–13]. We think that it is likely that women aged 19–24 may be less concerned or less encouraged to follow-up with further diagnostic testing than women in older age groups.

In our study, presenting symptoms at initial examination and subsequent referral was a significant predictor of completing an FNAC but not for an USS or MMG. An FNAC is an invasive procedure, and therefore, patients presenting with symptoms may be more inclined to complete the procedure. If population screening is to be scaled up, our study suggests a need for increased patient education around symptoms related to breast cancer and understanding the importance of completing diagnostic testing independent of symptoms.

A study conducted in Malawi showed that symptoms affected the behavior of seeking care. Patients delayed seeking care for their breast condition until the symptoms became painful [14] [15] [16],., A study conducted in Nigeria revealed many patients, whose breast screening revealed a breast mass and delayed seeking care because patients did not associate this symptom with a severe illness [17].

In our study, the odds of completing an MMG was higher if a patient attained at least a tertiary education. The education level was not found to be a predictor of FNAC or USS. Unlike the USS and FNAC, which are completed at ORCI, the mammogram test is completed off site, at either MNH or a different healthcare facility. This finding suggests that a higher level of education may be required to navigate through multisite processes and institutions and further supports the recommendation for a patient navigation program and that ORCI moves towards housing the entire diagnostic and treatment processes. Research investigating barriers to early presentation and diagnosis among women living in sub-Saharan Africa found insufficient knowledge of causes, symptoms, and breast cancer treatment among the study participants [18]. A study conducted in Egypt among breast cancer patients at a university setting in Cairo found that lower levels of awareness were related to lower levels of education [19]. More than half of the patients in our study had primary or no education. Therefore, increased education about breast cancer, screening, and the reasons for diagnostic testing is needed [20, 21].

Regarding the third observation of the lack of statistical significance of insurance status and residence for a diagnostic referral, it is essential to note a few points. First, while the Tanzanian government insurance covers treatment for low-income cancer patients, it does not cover diagnostic testing. Therefore, having government insurance does not mean much regarding diagnostic testing as it does not pay for diagnostic testing. Most patients have government insurance and pay out of the pocket for the diagnostic testing until they receive free treatment. Our results also showed that the place of residence did not have a statistically significant relationship to completing diagnostic testing. Previous research found that the increased travel time to health providers was associated with patient delays at the initial visit and between initial presentation to diagnosis and starting treatment [22]. Our study did not find an association between living close to the breast cancer screening program in Dar es Salaam and completion of diagnostic testing for any of the three tests. The lack of association may be related to the fact that most patients in our study (75% among all three diagnostic tests) lived in the same region: Dar es Salaam.

Our study has several key strengths, including a large sample size which increased the accuracy of the results. Additionally, patient demographics, referral information, and results of diagnostic testing were all stored in a centralized electronic medical record system for most patients included in the study [23].

We collected the data of the study several months after the end of 2018 to allow patients time to return after diagnosis outside ORCI. However, it is possible that some patients had diagnostic investigations and or even surgical treatment at MNH or other private centers but did not return to ORCI and therefore were coded as “no test completed.” Additionally, not all MMG results are documented in the chart when patients return to ORCI with their results. There may have been more patients who completed the test, but either did not return to ORCI with the results or the results were not documented in the medical records. Finally, a new visit was not always started in the electronic medical. This may have impacted the accuracy of the dates recorded for a CBE or diagnostic test, making the observed average lower than the actual average.

In summary, this study showed that about half of the referrals for further diagnostic testing did not return with diagnostic test results. The study also showed that older age, having symptoms at the initial appointment, and education were predictors of completing the referred diagnostic testing. The results of this study can inform policies that impact the availability of resources and the development of infrastructure for detection and diagnostic testing for breast cancer and contribute to our understanding of delays of diagnosis.

Future research should investigate the other diagnostic testing outside ORCI to verify if patients completed confirmatory diagnostic results elsewhere. Research should also focus on interviewing patients who completed and did not complete the recommended diagnostic referral. The interviews may elicit information not included in the medical records about their understanding of the recommended referral and other factors that may have influenced the decision to complete or not complete the referral diagnostic pathway [24, 6]. Examples of those factors may include financial ability to pay for diagnostic testing, waiting time at the diagnostic facilities, perceived seriousness of the condition, among other socioeconomic and behavioral patient factors, or health care system barriers. Future research should also investigate the cost effectiveness of housing all diagnostic and treatment services at ORCI and setting up a patient navigation program to follow patients to ensure diagnosis and necessary treatment completion.

## Figures and Tables

**Table 1:** Descriptive characteristics of patients who did or did not complete USS testing.

Ultrasound (USS)			
Variables	Completed test (%)	No test completed (%)	*p*
Education^∗^			
None or primary	76 (41.8)	81 (50.3)	0.2677
Secondary	61 (33.5)	44 (27.3)	
Tertiary	45 (24.7)	36 (22.4)	
Symptoms			
No	49 (21.8)	37 (20.1)	0.7717
Yes	176 (78.2)	147 (79.9)	
Lives in Dar Es Salaam			
No	44 (19.6)	43 (23.4)	0.4144
Yes	181 (80.4)	141 (76.6)	
Insurance			
Cash private	158 (70.2)	144 (78.3)	0.1896^∗∗∗^
NHIF	61 (27.1)	37 (20.1)	
Exempted	6( 2.7)	3 (1.6)	
Age^∗∗^ (19+)			
19–24	69 (31.2)	32 (17.5)	0.0001426
25–34	57 (25.8)	63 (34.4)	
35–44	47 (21.3)	47 (25.7)	
45–54	36 (16.3)	16 (8.7)	
55+	12 (5.4)	25 (13.7)	

^∗^66 patients referred for an USS did not have education data available. ^∗∗^5 patients did not have age data available. ^∗∗∗^Fisher exact test used for the calculation.

**Table 2:** Descriptive characteristics of patients who did or did not completed MMG testing.

Mammogram (MMG)			
Variables	Completed test (%)	No test completed (%)	*p*
Education^∗^			
None or primary	23 (71.9)	43 (65.2)	0.7266^∗∗∗^
Secondary	5 (15.6)	10 (15.2)	
Tertiary	4 (12.5)	13 (19.7)	
Symptoms			
No	1 (2.5)	10 (12.8)	0.09557^∗∗∗^ (Fisher exact)
Yes	39 (97.5)	68 (87.2)	
Lives in Dar es Salaam			
No	9 (22.5)	28 (35.9)	0.2022
Yes	31 (77.5)	50 (64.1)	
Insurance			
Cash private	22 (55.0)	48 (61.5)	0.6024^∗∗∗^
NHIF	17 (42.5)	29 (37.2)	
Exempted	1 (2.5)	1 (1.3)	
Age^∗∗^ (30+)			
30–44	10 (25.0)	13 (17.1)	0.5563
45–54	17 (32.5)	33 (43.4)	
55+	1 3(32.5)	30 (39.5)	

^∗^20 patients referred for an MMG did not have education data available. ^∗∗^2 patients did not have age data available. ^∗∗∗^Fisher exact test was used for the calculation.

**Table 3:** Descriptive characteristics of patients who did or did not complete FNAC testing.

Fine needle aspiration & cytology (FNAC)			
Variables	Completed test (%)	No test completed (%)	*p*
Education^∗^			
None or primary	92 (51.7)	43 (56.6)	0.6956
Secondary	48 (27.0)	20 (26.7)	
Tertiary	38 (21.3)	13 (17.1)	
Symptoms			
No	71 (33.0)	20 (20.4)	0.03391, <0.05
Yes	145 (67.0)	78 (79.6)	
Lives in Dar es Salaam			
No	61 (28.4)	16 (16.3)	0.0313, <0.05
Yes	154 (71.6)	82 (83.7)	
Insurance			
Cash private	141 (65.6)	77 (78.6)	0.06679
NHIF	51 (23.7)	15 (15.3)	
Exempted	23 (10.7)	6 (6.1)	
Age^∗∗^ (19+)			
19–24	37 (17.4)	22 (22.7)	0.2583
25–34	40 (18.8)	25 (25.8)	
35–44	55 (25.8)	20 (20.6)	
45–54	42 (18.7)	19 (19.6)	
55+	39 (18.3)	11 (11.3)	

^∗^59 patients referred for an FNAC did not have education data available. ^∗∗^3 patients did not have age data available.

**Table 4:** Crude and adjusted odds ratio and 95% confidence intervals of patients who did and did not complete USS testing.

Ultrasound (USS)							
Variables	Completed test (%)	No test completed (%)	Total referred for USS (%)	OR (crude)	CI (95%)	OR (adjusted)	CI (95%)
Education^∗^							
None or primary	76 (41.8)	81 (50.3)	157 (45.8)	1	NA	NA	NA
Secondary	61 (33.5)	44 (27.3)	105 (30.6)	0.68	0.41-1.12	0.76	0.43-1.31
Tertiary	45 (24.7)	36 (22.4)	81 (23.6)	0.75	0.44-1.29	1.06	0.56-2.01
Symptoms							
No	49 (21.8)	37 (20.1)	86 (21.0)	1	NA	NA	NA
Yes	176 (78.2)	147 (79.9)	323 (79.0)	1.11	0.68-1.80	1.3	0.76-2.24
Lives in Dar es Salaam							
No	44 (19.6)	43 (23.4)	87 (21.3)	1	NA	NA	NA
Yes	181 (80.4)	141 (76.6)	322 (78.7)	0.79	0.50-1.29	0.7	0.40-1.24
Insurance							
Cash private	158 (70.2)	144 (78.3)	302 (73.8)	1	NA	NA	NA
NHIF	61 (27.1)	37 (20.1)	98 (24.0)	0.67	0.42-1.06	0.62	0.34-1.13
Exempted	6 (2.7)	3 (1.6)	9 (2.2)	0.56	0.11-2.25	0.49	0.09-2.46
Age^∗∗^ (19+)							
19–24	69 (31.2)	32 (17.5)	101 (25.0)	1	NA	NA	NA
25–34	57 (25.8)	63 (34.4)	120 (29.7)	2.37	1.37-4.15	3.03	1.65-5.64
35–44	47 (21.3)	47 (25.7)	94( 23.3)	2.15	1.20-3.87	2.27	1.17-4.48
45–54	36 (16.3)	16 (8.7)	52 (12.9)	0.96	0.46-1.97	1.09	0.46-2.54
55+	12 (5.4)	25 (13.7)	37 (9.2)	4.41	1.10-10.24	4.01	1.66-10.11

^∗^66 patients referred for an USS did not have education data available. ^∗∗^5 patients did not have age data available.

**Table 5:** Crude and adjusted odds ratio for patients who did and did not complete MMG testing.

Mammogram (MMG)							
Variables	Completed test (%)	No test completed (%)	Total referred for MMG (%)	OR (crude)	CI (95%)	OR (adjusted)	CI (95%)
Education^∗^							
None or primary	23 (71.9)	43 (65.2)	66 (67.3)	1	NA	NA	NA
Secondary	5 (15.6)	10 (15.2)	15 (15.3)	1.05	0.33-3.84	1.34	0.35-5.60
Tertiary	4 (12.5)	13 (19.7)	17 (17.3)	1.69	0.52-6.77	4.29	1.05-21.00
Symptoms							
No	1 (2.5)	10 (12.8)	11 (9.3)	1	NA	NA	NA
Yes	39 (97.5)	68 (87.2)	107 (90.7)	0.2	0.01-1.11	0.307	0.02-2.04
Lives in Dar es Salaam							
No	9 (22.5)	28 (35.9)	37 (31.4)	1	NA	NA	NA
Yes	31 (77.5)	50 (64.1)	81 (68.6)	0.53	0.21-1.24	0.42	0.43-3.57
Insurance							
Cash private	22 (55.0)	48 (61.5)	70 (59.3)	1	NA		
NHIF	17 (42.5)	29 (37.2)	46 (39.0)	0.78	0.01-18.67	0.38	0.12-1.16
Exempted	1 (2.5)	1 (1.3)	2 (1.7)	0.46	0.36-1.73	0.33	0.01-9.07
Age^∗∗^ (30+)							
30–44	10 (25.0)	13 (17.1)	23 (19.8)	1	NA	NA	NA
45–54	17 (32.5)	33 (43.4)	50 (43.1)	1.49	0.53-4.15	1.53	0.42-5.49
55+	13 (32.5)	30 (39.5)	43 (37.1)	1.8	0.30-0.28	2.53	0.65-10.41

^∗^20 patients referred for an MMG did not have education data available. ^∗∗^2 patients did not have age data available.

**Table 6:** Crude and adjusted odds ratio for patients who did or did not complete FNAC testing.

Fine needle aspiration & cytology (FNAC)							
Variables	Completed test (%)	No test completed (%)	Total referred for FNAC (%)	OR (crude)	CI (95%)	OR (adjusted)	CI (95%)
Education^∗^							
None or primary	92 (51.7)	43 (56.6)	135 (53.1)	1	NA	NA	NA
Secondary	48 (27.0)	20 (26.7)	68 (26.8)	0.89	0.47-1.68	0.65	0.31-1.32
Tertiary	38 (21.3)	13 (17.1)	51 (20.1)	0.74	0.35-1.50	0.69	0.28-1.64
Symptoms							
No	71 (33.0)	20 (20.4)	91 (29.1)	1	NA	NA	NA
Yes	145 (67.0)	78 (79.6)	222 (70.9)	1.91	1.10-3.45	1.91	1.02-3.72
Lives in Dar es Salaam							
No	61 (28.4)	16 (16.3)	77 (24.6)	1	NA	NA	NA
Yes	154 (71.6)	82 (83.7)	236 (75.4)	2.01	1.11-3.83	1.55	0.76-3.32
Insurance							
Cash private	141 (65.6)	77 (78.6)	217 (69.3)	1	NA	NA	NA
NHIF	51 (23.7)	15 (15.3)	66 (21.1)	0.54	0.28-1.01	0.49	0.21-1.10
Exempted	23 (10.7)	6 (6.1)	2 9(9.3)	0.49	0.17-1.19	0.56	0.15-1.71
Age^∗∗^ (19+)							
19–24	37 (17.4)	22 (22.7)	59 (19.0)	1	NA	NA	NA
25–34	40 (18.8)	25 (25.8)	65 (21.0)	1.05	0.51-2.19	1.16	0.50-2.71
35–44	55 (25.8)	20 (20.6)	75 (24.2)	0.61	0.29-1.29	0.78	0.32-1.86
45–54	42 (18.7)	19 (19.6)	61 (19.7)	0.76	0.35-1.63	0.8	0.30-2.10
55+	39 (18.3)	11 (11.3)	50 (16.1)	0.48	0.20-1.12	0.56	0.19-1.58

^∗^59 patients referred for an FNAC did not have education data available. ^∗∗^3 patients did not have age data available.

## Data Availability

The data is stored at the Ocean Road Cancer Institute in Tanzania.
